# Using machine learning methods to predict post-traumatic stress disorder in stroke patients in China

**DOI:** 10.3389/fpsyt.2025.1694654

**Published:** 2025-11-20

**Authors:** Ying Li, Chuang Pan, Yue Gu

**Affiliations:** 1 College of Sports Science, Jishou University, Jishou, Hunan, China; 2 School of Nursing, Guilin Medical University, Guilin, Guangxi, China; 3 Operating Room, Taizhou People’s Hospital Affiliated to Nanjing Medical University, Taizhou, Jiangsu, China

**Keywords:** stroke, post-traumatic stress disorder, random forest, prediction model, machine learning, risk factors

## Abstract

**Background:**

This study aims to utilize various machine learning algorithms to construct a risk prediction model for post-stroke Post-Traumatic Stress Disorder (PTSD), select the optimal model, and identify risk factors.

**Methods:**

A total of 249 stroke patients from two tertiary hospitals in Jiangsu Province and Shandong Province were selected and randomly divided into the training group and the validation group. Based on the results of Logistic regression analysis, a risk prediction model for PTSD after stroke was constructed by using Logistic regression, Random forest (RF) and K-nearest neighbor algorithm, and further verification was conducted according to the best algorithm.

**Results:**

The incidence of PTSD in stroke patients was 40.56%, and the RF model was the best. Feature importance ranking shows that the factors affecting PTSD in stroke patients are: Stroke type (0.187), Sleep in the last three months (0.152), Way of hospitalization (0.147), Monthly household income (0.133), Hypertension (0.108), Gender (1.104), Marital status (0.079), Physical exercise situation (0.067), and Educational background (0.023).

**Conclusion:**

The model based on the RF algorithm has the best predictive performance, and the factors affecting PTSD in stroke patients include stroke type, gender, Way of hospitalization, Sleep in the last three months, Physical exercise situation, Hypertension, etc. The results of this study can assist clinical medical staff to screen high-risk groups of PTSD after stroke and provide the basis for early implementation of targeted preventive measures.

## Introduction

Post-traumatic stress disorder (PTSD) is an anxiety disorder triggered by exposure to traumatic events, characterized by re-experiencing the event, avoidance of reminders, persistent negative emotions and cognitions, and physiological hyperarousal that persists for at least one month after the event (1). Stroke is a severe disease and a leading cause of death and long-term disability, characterized by high incidence, disability, mortality, recurrence, and economic burden, imposing a significant global burden (2). The occurrence of stroke presents substantial risks and negative impacts on patients’ physical and mental health (3), frequently leading to the development of PTSD (4, 5), with an incidence rate ranging from 3% to 37% among stroke patients (1, 6, 7). Following the onset of PTSD in stroke patients, medication compliance typically decreases (8), resulting in elevated levels of tension, fear, sleep disturbances, and the emergence of post-stroke fatigue (PSF), which impairs recovery and reduces the likelihood of returning to work (9),significantly diminishing the quality of life (10).Although conventional statistical methods like regression analysis have identified risk factors and provided foundational insights, their predictive validity is substantially limited in post-stroke PTSD populations (4, 11). This limitation stems from the condition’s complex, multifactorial etiology, which involves substantial heterogeneity and intricate nonlinear interactions that traditional models fail to capture (12). Therefore, developing predictive tools for early screening, identifying risk factors for stroke-related PTSD, and implementing early interventions are crucial to halt PTSD progression and mitigate its harm. Machine learning has emerged as a robust tool for risk prediction modeling, leveraging its powerful data mining and processing capabilities and proficiency in handling nonlinear relationships between complex variables (13). Although machine learning has been applied to identify risk factors for post-stroke PTSD, existing research has predominantly relied on individual models. This approach creates a critical gap, as the absence of a comprehensive comparative analysis prevents the determination of the most effective predictive model. Although machine learning has been applied to identify risk factors for post-stroke PTSD, existing research has predominantly relied on individual models (14, 15). This approach creates a critical gap, as the absence of a comprehensive comparative analysis prevents the determination of the most effective predictive model. In recent years, the random forest (RF) algorithm has been widely used in disease risk prediction, early warning, and prognosis, while logistic regression enables quantitative analysis and intuitive explanation of influencing factors through OR value (16). K-Nearest Neighbors (KNN) detects local patterns in demographically similar individuals. This study employed logistic regression, RF, and KNN to analyze the status and influencing factors of PTSD in stroke patients from two tertiary hospitals. The objective was to develop a predictive model to identify high-risk individuals and provide a scientific basis for preventing PTSD in this population.

## Methods

### Participants

A total of 249 patients diagnosed with stroke from two tertiary hospitals in Jiangsu Province and Shandong Province between October 2023 and December 2023 were selected as the study population. In accordance with the Declaration of Helsinki, all subjects gave written informed consent (17). The inclusion criteria for this study were as follows: Patients included in the study met the diagnostic criteria for ischemic stroke (18), with initial confirmation through CT and/or MRI imaging. Additionally, they met the diagnostic criteria for hemorrhagic stroke (19);Inclusion criteria also stipulated an age of ≥18 years, clear consciousness, and voluntary participation in the research. Exclusion criteria were: Patients with severe diseases of the heart, brain, kidneys, or other organ systems were excluded from the study. Additionally, individuals with speech or cognitive impairments, as well as those with psychiatric disorders or intellectual disabilities, were not eligible for participation.

### General information and questionnaire data

Our study comprised two main components. Our research consists of two main parts. The first part includes general demographic information, a total of 28 items, including gender, age, living style, place of residence, family income, marital status, hypertension (Meet the diagnostic criteria for hypertension established by the Chinese Guidelines for the Prevention and Treatment of Hypertension (2024 Revision) (20)), diabetes (meet the diagnostic criteria for diabetes (21)), etc. The Impact of Event Scale-Revised (IES-R) (22) was utilized to assess the intrusiveness, avoidance, or hyperarousal thoughts related to the health event. IES-R was revised by Weiss (23) and Marmar (24) in 1997, and it was translated into Chinese by the Chinese scholar Guo Suran as early as 2007 (25).This scale comprises 22 items, with patients rating the severity of their distress regarding cancer and its treatment over the past week for each item on a scale of 0 (never) to 4 (always), resulting in a total score ranging from 0 to 88. Higher scores indicate more pronounced post-traumatic stress reactions, with a score of ≥35 suggesting a positive PTSD screening (26). In this study, the Cronbach’s α for the sample was 0.953.

### Data collection

Prior to commencing the survey, all investigators underwent standardized training via video sessions, covering survey principles, procedural precautions, and guidance on clear explanations. During the survey, investigators engaged patients in face-to-face interactions, presenting paper-based questionnaires to elucidate the survey’s purpose, content, and completion process. Upon obtaining patient consent, questionnaires were distributed, allowing patients to independently complete them. Completed questionnaires were promptly retrieved on-site. In instances where patients were unable to complete the questionnaire due to unique circumstances, investigators impartially presented the questions and assisted in truthful completion. A total of 260 questionnaires were distributed, with 249 effectively collected, yielding a robust retrieval rate of 95.7%.

### Statistical analysis

Paper questionnaire was used to collect data, and Excel was used to organize the data. SPSS 27.0 software was used for data analysis. Data that did not meet our study inclusion and exclusion criteria were deleted. Disaggregated data are expressed in frequency and percentage terms. A Python script is used to shuffle the data and distribute it between the training set (70%) and the verification set (30%). Chi-square test and Mann-Whitney U test were used for general data comparison, and logistic regression was used for risk factor screening. Logistic regression, RF and K-nearest neighbor algorithm were used to construct a cognitive impairment risk prediction model for stroke patients in training concentration. The optimal hyperparameters were obtained by grid search and 5-fold cross-validation, and the optimal threshold was determined by Jorden index. Validation sets were used to evaluate the model’s performance, including area under the curve (AUC), sensitivity, specificity, and Jorden index. When the results of each index are inconsistent, the AUC is used as the main reference, and the evaluation results of the validation set are used as the basis for selecting the best model.

## Results

### General characteristics of PTSD in stroke patients

A total of 249 stroke patients were surveyed in this study, comprising 134 males and 115 females. Among them, 101 individuals (40.56%) were diagnosed with PTSD, while 148 individuals (59.44%) did not exhibit PTSD symptoms. Thirty-three potential risk factors were evaluated, and a comparison of general characteristics between the train groups and test groups is presented in **Table 1**.

**Table 1:** Clinical characteristics of patients.

Variable	Total	No	Yes	χ²/Z	P
Gender				10.228	0.001**
Male	134	92	42		
Female	115	56	59		
Age(Years)				1.791	0.617
≤60	60	32	28		
61-70	84	54	30		
71-80	87	51	36		
≥81	18	11	7		
Educational background				15.519	<0.001***
High school education or below	221	141	80		
College or undergraduate	28	7	21		
Place of Residence				1.626	0.202
Countryside	87	47	40		
City	162	101	61		
Living style				5.705	0.127
Nursing home	6	3	3		
Live alone	41	19	22		
Live with spouse	164	106	58		
Live with children	38	20	18		
Marital status				18.077	<0.001**
Unmarried	13	5	8		
Married	196	129	67		
Divorce	24	6	18		
Widowed	16	8	8		
Monthly household income				13.410	0.004*
< 1000 RMB	28	10	18		
1000–2000 RMB	59	36	23		
2000–4000 RMB	93	51	42		
≥4000 RMB	69	51	18		
Type of medical insurance				7.214	0.065
Urban medical insurance	122	64	58		
Urban and rural medical insurance	120	80	40		
self-financing	5	2	3		
Other type	2	2	0		
Stroke type				43.326	<0.001**
Hemorrhagic Stroke	41	18	23		
Ischemic Stroke	155	116	39		
Other type	53	14	39		
Cerebrovascular history				0.978	0.323
No	184	106	78		
Yes	65	42	23		
Fall history in the last 6 months					
Yes	36	24	12	0.912	0.340
No	213	124	89		
Physical exercise situation					
Exercise every day (≥30min per day)	64	39	25	12.701	0.002*
Occasionally (1–2 times/week)	97	69	28		
No exercise	88	40	48		
Sleep in the last three months				13.966	<0.001**
Good	96	43	53		
Average	98	68	30		
Poor	55	37	18		
Smoking				0.008	0.927
Yes	166	99	67		
No	83	49	34		
Drinking history				0.012	0.914
Yes	184	109	75		
No	65	39	26		
Number of lesions				3.482	0.062
2 or less	244	143	101		
3 or more	5	5	0		
Diabetes				1.025	0.311
Yes	171	98	73		
No	78	50	28		
Hypertension				19.048	<0.001**
No	113	84	29		
Yes	136	64	72		
Coronary heart disease				0.003	0.957
Yes	20	12	8		
No	229	136	93		
Chronic kidney disease				0.858	0.354
Yes	3	1	2		
No	246	147	99		
Cerebral atrophy				4.288	0.038
Yes	225	129	96		
No	24	19	5		
Multiple cerebral infarction				4.202	0.040*
Yes	216	123	93		
No	33	25	8		
Cerebral protein deficiency				0.430	0.512
Yes	242	143	99		
No	7	5	2		
Carotid atherosclerotic plaque				0.599	0.439
Yes	184	112	72		
No	65	36	29		
Atherosclerosis of the carotid artery				0.713	0.399
No	170	98	72		
Yes	79	50	29		
Sleep in the last three months				13.966	<0.001***
Good	55	37	18		
Average	98	68	30		
Poor	96	43	53		
Way of hospitalization				27.727	<0.001***
Emergency admission	115	56	59		
Outpatient admission	107	83	24		
Ward transfer	27	9	18		
Physical exercise situation				12.701	0.002**
Exercise regularly	64	39	25		
Exercise occasionally	97	69	28		
Do not exercise	88	40	48		

*P<0.05, **P<0.01, P<0.001.

### PTSD status in stroke patients

The results of this study revealed that the total score of PTSD in stroke patients was 29.12 ± 15.932, with the intrusion dimension scoring the highest (10.98 ± 5.79) and the hyperarousal dimension scoring the lowest (7.83 ± 4.99). Detailed scores are presented in **Table 2**.

**Table 2:** Total PTSD scores and dimensions in stroke patients.

Items	Score
Avoidance Dimension	10.30 ± 6.11
Intrusion Dimension	10.98 ± 5.79
Hyperarousal Dimension	7.83 ± 4.99
Total Score	29.12 ± 15.93

### Factors associated with PTSD: logistic regression analysis

In the training data set, the occurrence of PTSD after stroke was taken as the dependent variable (No=0, Yes=1). **Table 1** shows Gender (Male=0, Female=1), Educational background(High school education or below=0, College or undergraduate=1), Marital status(Married=0, Unmarried=1, Divorce=2, Widowed=3), Monthly household income(≥4000 RMB = 0, 2000–4000 RMB = 1, 1000–2000 RMB = 2, < 1000RMB = 3), Hypertension (No=0, Yes=1), Sleep in the last three months(Good=0, Average =1, Poor=2),Way of hospitalization (Outpatient admission=0, Emergency admission=1, Ward transfer=2), Physical exercise situation(Do not exercise=0, Exercise regularly=1, Exercise occasionally=2), Stroke type(Ischemic Stroke=0, Hemorrhagic Stroke=1, Other type=2), Multiple cerebral infarction(No=0, Yes=1) were independent variables and logistic regression analysis was performed. A total of 9 risk factors were found through analysis (all P < 0.05), as shown in **Table 3**.

**Table 3:** Logistic regression analysis of post-stroke PTSD.

Risk factor	Reference factor	B	SE	Waldx^2^	P	OR	95%CI
Gender	Male						
Female		0.836	0.264	10.066	0.002	2.308	1.377 to 3.869
Educational background	High school education or below	1.665	0.458	13.202	<0.001	5.287	2.153 to 12.983
College or undergraduate							
Marital status	Married	1.754	0.495	12.559	<0.001	5.776	2.190 to 15.236
Divorce							
Monthly household income	≥4000 RMB						
<1000 RMB		1.629	0.480	11.505	<0.001	5.100	1.989 to 13.075
Stroke type	Ischemic Stroke	1.335	0.365	13.374	<0.001	3.801	1.858 to 7.774
Hemorrhagic Stroke		2.115	0.362	34.046	<0.001	8.286	4.071 to 16.858
Other type							
Hypertension	No						
Yes		1.181	0.276	18.385	<0.001	3.259	1.899 to 5.592
Sleep in the last three months	Good						
Poor		0.930	0.353	6.930	0.008	2.534	1.268 to 5.062
Way of hospitalization	Outpatient admission						
Ward transfer		1.293	0.298	18.886	<0.001	3.644	2.034 to 6.528
Emergency admission		1.934	0.469	16.971	<0.001	6.917	2.756 to 17.358
Physical exercise situation	Do not exercise						
Exercise regularly		-1.084	0.310	12.240	<0.001	0.338	0.184 to 0.620

### Construction and validation of a predictive model for PTSD risk in stroke patients

**Figure 1:**
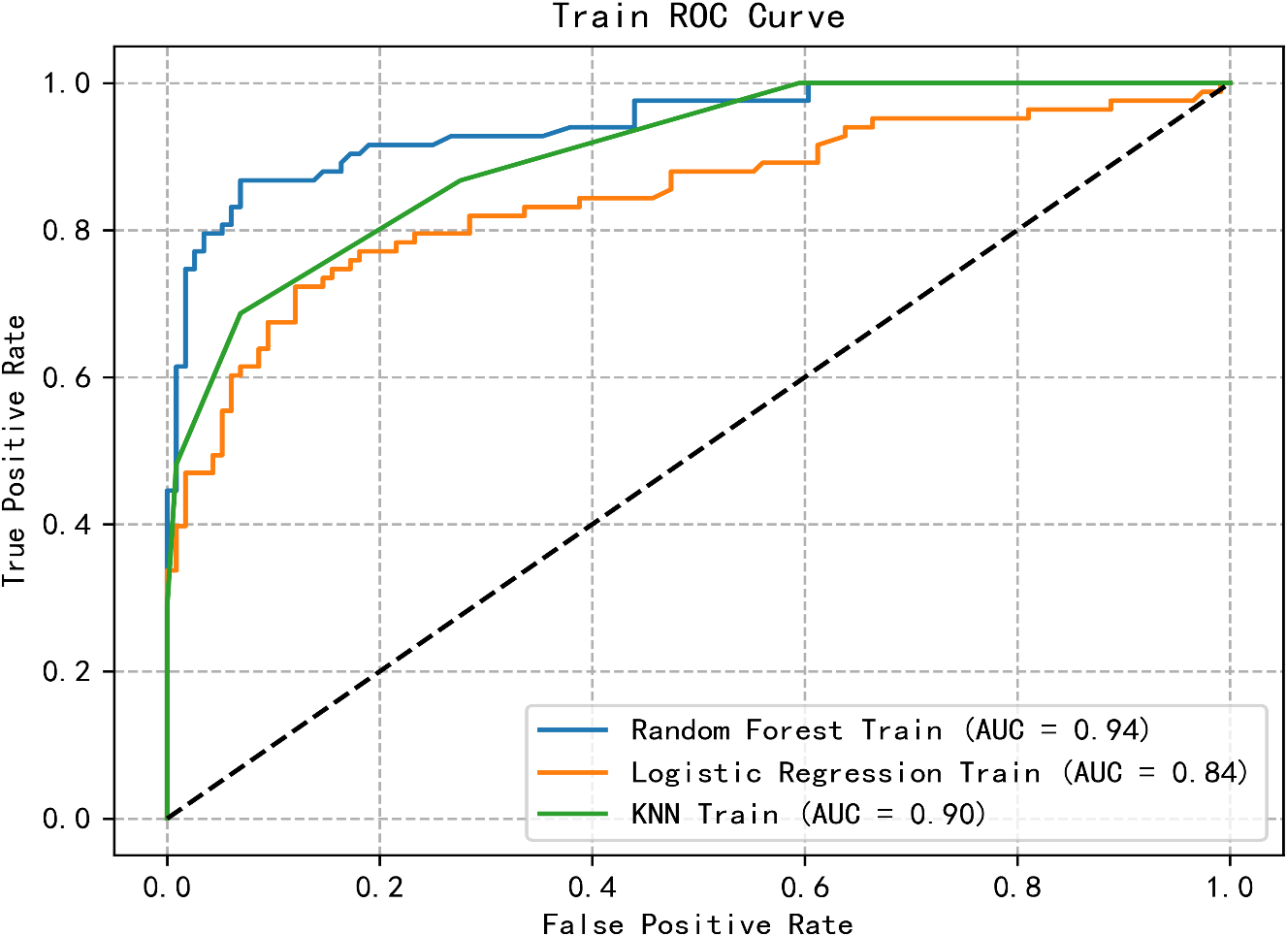
ROC curve of the training group of three machine learning algorithms.

**Figure 2:**
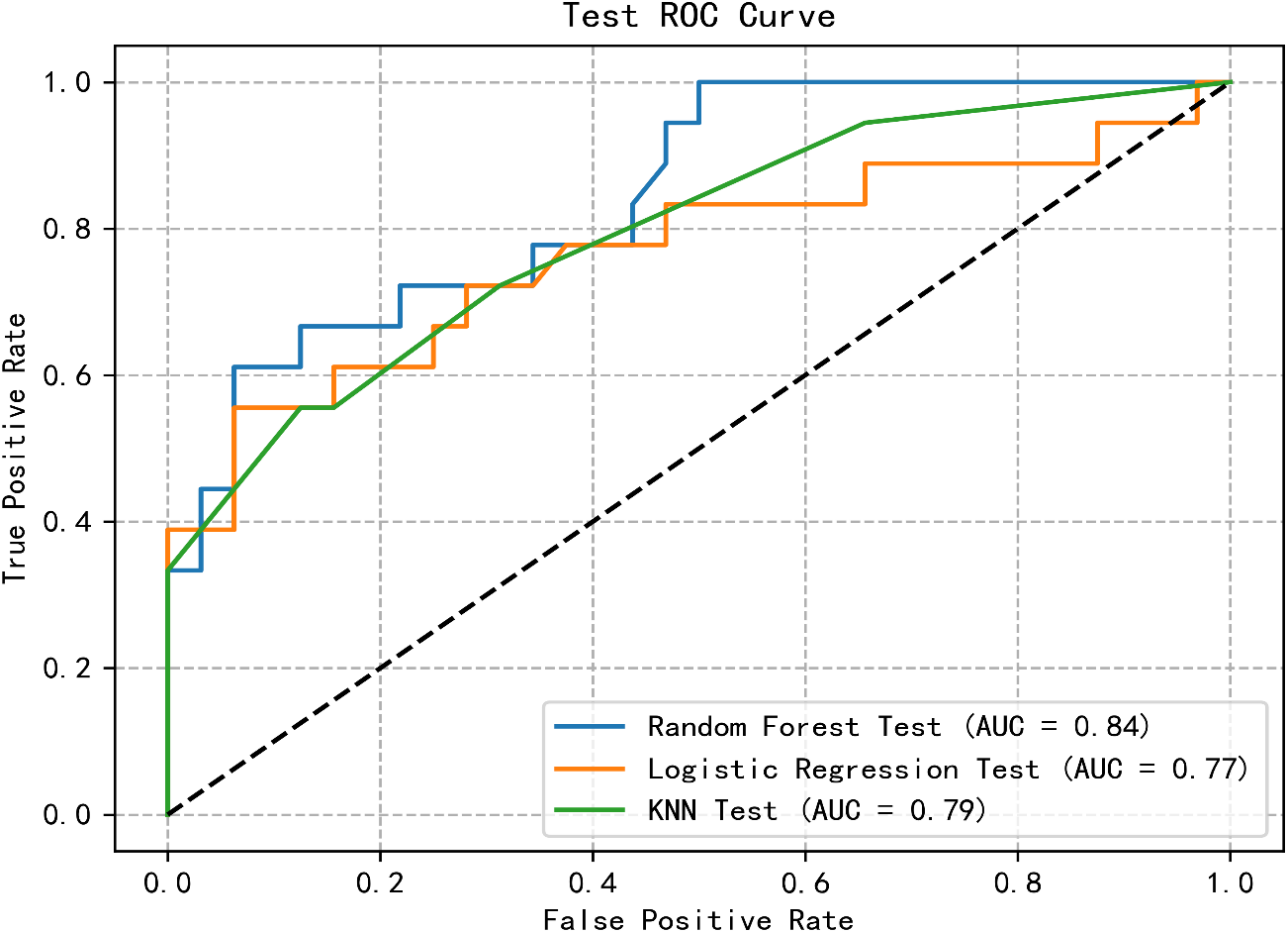
ROC curve of test group of three machine learning algorithms.

The predictive model for PTSD risk in stroke patients was constructed and validated in this study. Utilizing the PyCharm software, the scikit-learn library was employed to implement logistic regression, RF, and k-nearest neighbor algorithms. Data from both the training and validation sets were imported, with six influencing factors as independent variables and the occurrence of PTSD as the dependent variable. The models were trained, and optimal parameters were determined through grid search and 5-fold cross-validation. Subsequently, the models were validated using the validation set, and metrics including AUC, sensitivity, specificity, and Youden’s index were calculated for each model. Results indicated that the logistic regression model had the lowest AUC, while the RF model exhibited the highest AUC. Additionally, the RF model demonstrated optimal sensitivity and precision, whereas the logistic regression model showed the lowest sensitivity and precision. Detailed results are presented in **Table 4**. The receiver operating characteristic (ROC) curves for the training and validation group are illustrated in **Figures 1**, **2**.

**Table 4:** Comparison of prediction models.

Prediction model	AUC	Accuracy	Sensitivity	Specificity	Recall	Precision	F1 Score
Random forest model training group	0.93	0.800	0.966	0.795	0.885	0.905	0.885
Random forest model validation group	0.84	0.895	0.875	0.667	0.775	0.785	0.78
Logistic model training group	0.84	0.76	0.844	0.611	0.80	0.815	0.81
Logistic model testing group	0.77	0.80	0.667	0.844	0.775	0,74	0.78
k-nearest neighbor training group	0.90	0.74	0.667	0.931	0.810	0.835	0.815
k-nearest neighbor testing group	0.79	0.74	0.556	0.844	0.7	0.72	0.71

### Feature importance ranking

**Figure 3:**
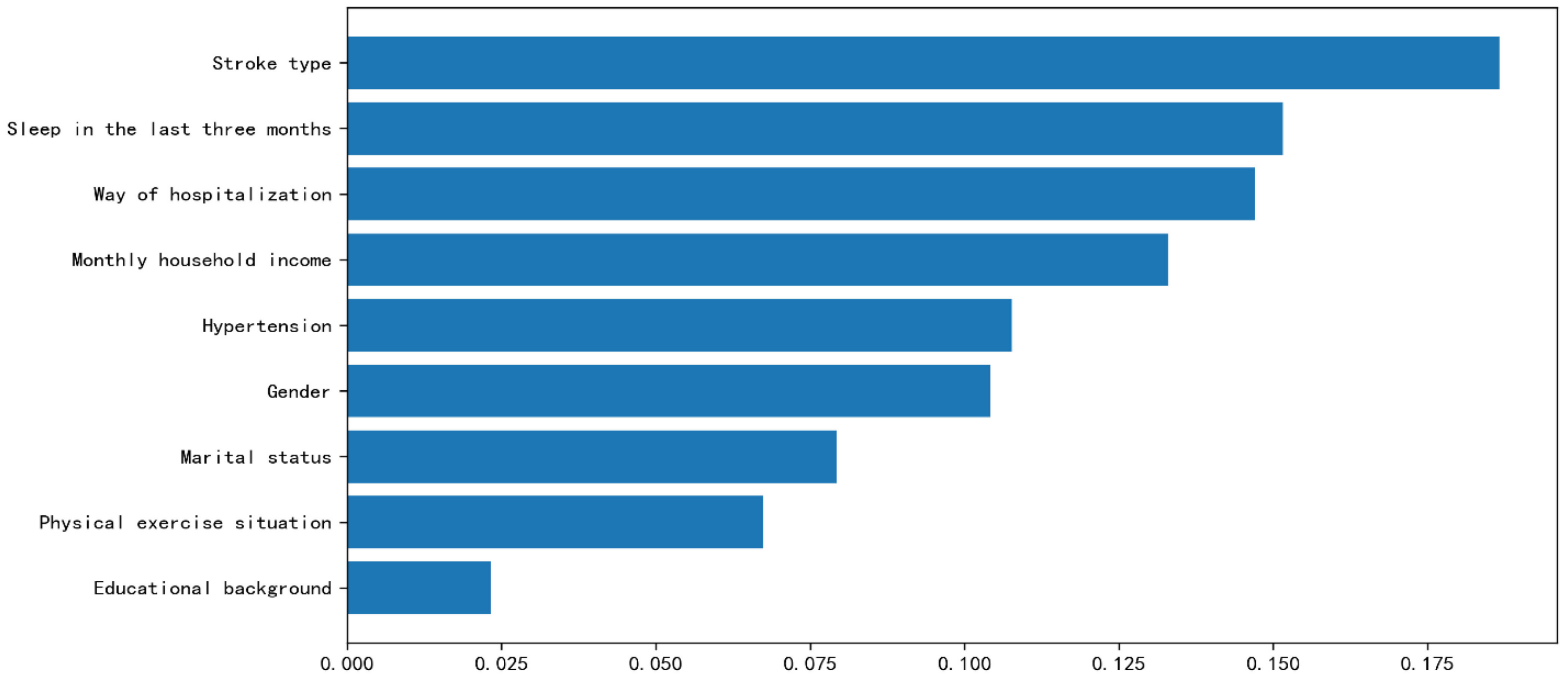
Feature importance ranking.

The factors affecting PTSD in patients with stroke are as follows: Stroke type (0.187), Sleep in the last three months (0.152), Way of hospitalization (0.147), Monthly household income (0.133), Hypertension (0.108), Gender (1.104), Marital status (0.079), Physical exercise situation (0.067), and Educational background (0.023) (**Figure 3**). The Brier scores of RF, Logistic, and KNN were 0.157, 0.179, and 0.179, respectively. At the same time, we also conducted further verification through Partial Dependency Plots (PDPs) (**Supplementary Figures 1-5**).

## Discussion

In this study, three machine learning algorithms—logistic regression, RF, and KNN—were employed to construct predictive models for PTSD symptoms. The results revealed that the RF model exhibited the highest values for the area under the ROC, specificity, sensitivity, and F1 score, indicating its superiority over the other models. The findings indicate that the RF model represents the optimal choice, while logistic regression demonstrated the least favorable overall performance among the models. Compared to alternative models, RF aggregates classification decisions from multiple trees, selecting the most frequently occurring class as the final output (27). Its ensemble-based nature provides a substantial advantage over traditional regression methods when handling nonlinear data. The RF model effectively addresses classification and regression tasks (28). The inherent randomness in the RF algorithm enhances its data fitting capability and provides robustness against noise and overfitting (29). To optimize the RF model’s performance, grid search was employed to optimize its hyperparameters, evaluating model performance through cross-validation of different hyperparameter combinations to select the optimal configuration. Feature importance analysis revealed each feature’s contribution to the classification process, identifying key factors affecting prediction results. Our RF model demonstrated favorable performance in screening for post-traumatic stress disorder among stroke survivors, achieving an area under the curve of 0.84. This represents a competitive advantage compared to prior studies (30), complemented by its satisfactory sensitivity, accuracy, and F1-score on internal validation. By identifying key predictor variables, the model also provides clinically interpretable insights, establishing a foundation for early detection and targeted interventions. Its balanced performance in case identification and false-positive reduction positions it as a suitable initial screener within a stepped-care framework. This approach allows for optimized resource allocation by focusing comprehensive diagnostic assessments on a pre-identified high-risk subgroup, while future clinical translation could involve calibrating the decision threshold to accommodate local healthcare needs.

The research findings indicate a post-stroke PTSD incidence of 40.56%, exceeding previous studies (31, 32). These discrepancies may stem from variations in geographical settings, diagnostic tools, ethnic demographics, and healthcare disparities (33). Post-stroke PTSD significantly impacts patients’ occupational and daily functioning, manifesting in severe somatic symptoms (34) and increased comorbidity and suicide risks (35). The study revealed the highest scores in the intrusion dimension (10.98 ± 5.79) and lowest in hyperarousal (7.83 ± 4.99). Intrusion dimension encompasses patients’ impressions and recollections of disease onset, progression, and treatment (36). Elevated intrusion scores correlate with increased anxiety tendencies, as patients exhibit disease-related apprehension and hypervigilance (37). Evidence indicates that PTSD not only increases cardiovascular disease risk (38) but also serves as an independent risk factor for stroke occurrence (39).Risk factor screening through logistic and RF model analysis revealed the following hierarchy of risk factors: Stroke type, Sleep in the last three months, Way of hospitalization, Monthly household income, Hypertension, Gender, Marital status, Physical exercise situation, and Educational background.

While existing research has explored risk factors for PTSD in stroke patients, the relationship between stroke type and PTSD remains largely unexplored (40). This study demonstrates that stroke type significantly influences PTSD development. Ischemic stroke occurs when blood supply to brain tissue is compromised due to vascular obstruction, while hemorrhagic stroke involves blood vessel rupture into the cerebral parenchyma or ventricle (41). Although hemorrhagic stroke has a lower incidence rate, it presents greater severity, mortality, disability rates, and economic burden compared to ischemic stroke. The distinct treatment and rehabilitation protocols for hemorrhagic stroke (42) may intensify patients’ psychological distress, potentially contributing to PTSD development. The study reveals that stroke patients with hypertension demonstrate increased PTSD risk compared to those without hypertension. The chronic nature and fluctuations of hypertension-induced intracerebral hemorrhage (43) heighten stroke patients’ anxiety. Furthermore, hypertension imposes substantial economic and medical burdens on patients’ families. Post-stroke activity restrictions and medication side effects compound patients’ psychological burden, potentially exacerbating PTSD symptoms.

Gender has been identified as a risk factor influencing PTSD in stroke patients, with females exhibiting a higher risk compared to males, consistent with previous research findings (44). Studies have shown that females tend to engage in more emotional expression behaviors than males. When faced with the same event, males typically express themselves in a cognitive-focused manner, while females tend to employ an emotion-focused approach (45, 46). The onset of stroke is often sudden and severe, leading patients to experience sudden changes in physical condition, bodily pain, functional impairments, and other stressors (47), Consequently, patients often resort to negative coping strategies such as refusal to comply with treatment, venting, and irritability, which predisposes females to a higher risk of developing PTSD (48). Additionally, differences in hippocampal structure between males and females are also cited as one of the factors contributing to the increased vulnerability of females to PTSD (49). Multiple studies have indicated that low educational background is a contributing factor to PTSD following stroke (47, 49, 50).Sufficient cultural literacy provides crucial support for interpersonal relationships, offering patients effective internal resources for problem-solving (51). Higher education levels typically correlate with better economic conditions and health literacy (52), enabling more effective utilization of social resources and support systems. These patients generally demonstrate better disease comprehension, reducing illness-related fears and facilitating psychological adaptation (53), thus lowering PTSD risk. Conversely, patients with limited education often struggle with disease understanding, emotional management, and problem-solving, increasing their PTSD susceptibility. Healthcare professionals should therefore consider patients’ information processing capabilities, particularly for female stroke patients and those with lower educational levels. They should provide guidance for appropriate disease understanding, minimize knowledge-related anxieties, and assist with emotional management to reduce PTSD incidence. Additionally, this study identifies marital status as an influential factor in stroke-related PTSD, ranking seventh in feature importance. These findings differ from Stela’s research (54). Studies indicate that interpersonal-oriented therapies, including couple and family interventions, may ameliorate PTSD symptoms, with positive intimate relationships serving as protective factors (55). Married individuals often benefit from partner support, enabling better psychological pressure management and emotional regulation, leading to improved overall health status and reduced post-stroke psychological distress (56). Divorced patients, experiencing emotional disconnection and lacking partner support, typically demonstrate poorer psychological states and increased susceptibility to post-stroke PTSD.

The findings of this study demonstrate that the mode of admission significantly influences PTSD occurrence in stroke patients, with emergency department admissions showing higher susceptibility to PTSD. Patients admitted through the emergency department typically experience acute onset stroke, in which severe cerebrovascular dysfunction develops suddenly, inducing substantial psychological distress manifested through anxiety, fear, and helplessness. The abrupt onset of illness provides these patients with limited time for adaptation and coping, thus elevating their PTSD risk. Additionally, the trauma-induced tension and panic activate the amygdala within the brain’s limbic system, a region crucial for forming conditioned fear memories, which constitute core PTSD symptoms (57). Healthcare providers treating stroke patients admitted through emergency departments should strengthen patient and family communication, implement proactive psychological counseling, and assist patients in developing positive attitudes and effective coping strategies.

Research identifies low household income as a risk factor for PTSD (58), aligning with previous study findings. The extended medical and rehabilitative care required by stroke patients creates financial pressure, potentially increasing anxiety and concern, thereby elevating the risk of stress disorders. Additionally, the prolonged recovery period following severe trauma reduces patients’ capacity, confidence, and motivation to resume work, intensifying economic challenges and exacerbating PTSD symptoms (59).For stroke patients with lower household incomes, clinical healthcare providers should actively monitor their psychological well-being, offer psychological support and care, assist them in facing their illness positively, alleviate psychological stress, and reduce the occurrence of PTSD.

Physical exercise demonstrates multiple beneficial effects: reducing stress and anxiety, facilitating neurological changes, activating neurotransmitters while regulating brain function, promoting neuronal growth (60), maintaining neuroplasticity, and enhancing mitochondrial activity in the brain, consequently improving PTSD symptoms (61). The research indicates that regular physical activity serves as a protective factor against PTSD in stroke patients. Physical inactivity among stroke patients may result in diminished physical function, substantially affecting their daily living capabilities and quality of life, while increasing future uncertainty, thereby elevating risks of anxiety, depression, and PTSD onset. This study also found that poor (or very poor) sleep quality among stroke patients is a risk factor for PTSD. Sleep quality issues are prevalent among stroke patients, with sleep disturbances being a common problem affecting their sleep quality (62). Sleep disturbances are crucial predictors for the development of PTSD, with both pre- and post-traumatic sleep disturbances increasing the risk of PTSD (63, 64). Additionally, research has found that sleep disturbances mediate the relationship between PTSD and suicidal ideation among hospitalized patients (65). The sleep condition of stroke patients should not be overlooked, and healthcare professionals should actively create a conducive sleep environment during hospitalization, assist patients in developing good sleep habits, avoid stimulating substances, and improve sleep quality to reduce the occurrence of PTSD.

## Strengths and limitations

This study constructed prediction models for PTSD in stroke patients using RF algorithm, logistic regression, and KNN machine learning approaches. The analysis demonstrated that the RF model exhibited superior predictive performance, providing valuable insights for clinical diagnosis, treatment, and nursing protocols. However, several limitations warrant consideration. First, as the study relied on patient self-reported data, certain potentially influential factors affecting PTSD incidence may be missing or incomplete, particularly physiological and biochemical parameters. Second, the absence of genomic information may limit mechanistic insights. Furthermore, the cross-sectional nature of the study and its relatively small sample size may impact the generalizability of the results. Future research would benefit from multi-center, large-scale studies, establishing comprehensive follow-up cohorts, and investigating additional objective and quantifiable predictors to validate these findings.

## Conclusion

This investigation employed three machine learning algorithms to develop a risk prediction model for post-traumatic stress disorder in stroke patients, with the RF model emerging as the most effective predictor. The study identified several risk factors for PTSD in stroke patients, including stroke type, physical exercise, sleep quality, gender, education level, and family economic status. These findings enable clinical medical staff to monitor risk factors for post-traumatic stress disorder in stroke patients and develop tailored intervention strategies to mitigate its impact. Given the cross-sectional design and limited sample size, which may not fully represent patients across different healthcare facilities and geographical regions, future large-scale, multi-center, longitudinal studies are essential. Furthermore, as machine learning continues to advance as a core technology in artificial intelligence, its robust data mining and processing capabilities show increasing promise in disease risk prediction. Future research should explore additional machine learning algorithms to enhance PTSD risk prediction models for stroke patients, thereby strengthening the foundation for effective screening and intervention strategies.

## Data Availability

The original contributions presented in the study are included in the article/**Supplementary Material**. Further inquiries can be directed to the corresponding author.
