# Supplementary figures and images for: Using machine learning methods to predict post-traumatic stress disorder in stroke patients in China
Source: Front Psychiatry. 2025 Nov 20;16:1694654. doi: 10.3389/fpsyt.2025.1694654 (PMC12675482; doi:10.3389/fpsyt.2025.1694654)

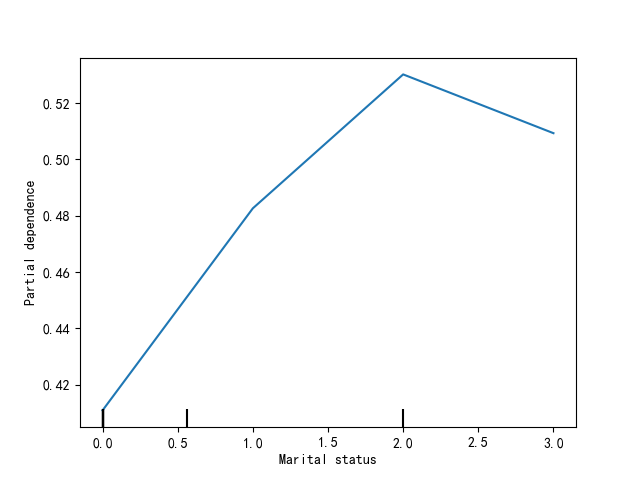

Supplement: Supplementary file 1 [file DataSheet1.zip › Supplementary Figure/Figure 1. Marital status.png]

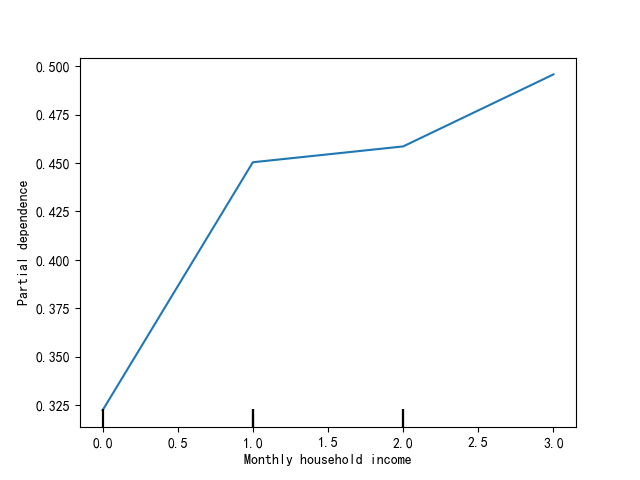

Supplement: Supplementary file 1 [file DataSheet1.zip › Supplementary Figure/Figure 2. Monthly household income.png]

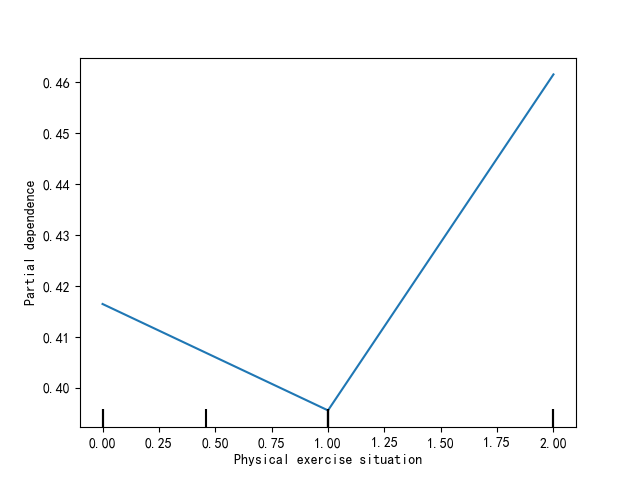

Supplement: Supplementary file 1 [file DataSheet1.zip › Supplementary Figure/Figure 3. Physical exercise situation.png]

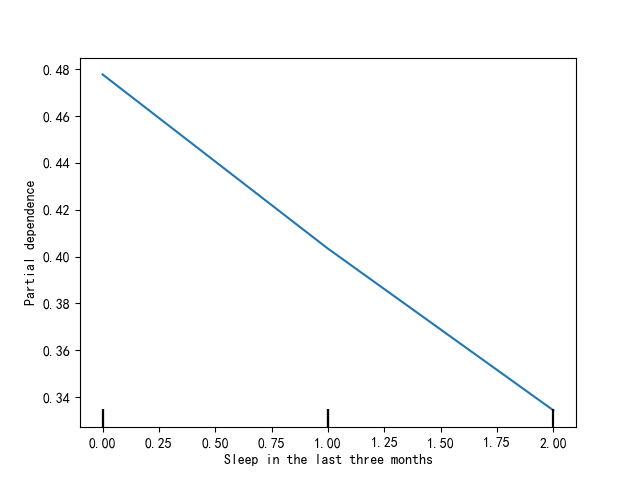

Supplement: Supplementary file 1 [file DataSheet1.zip › Supplementary Figure/Figure 4. Sleep in the last three months.png]

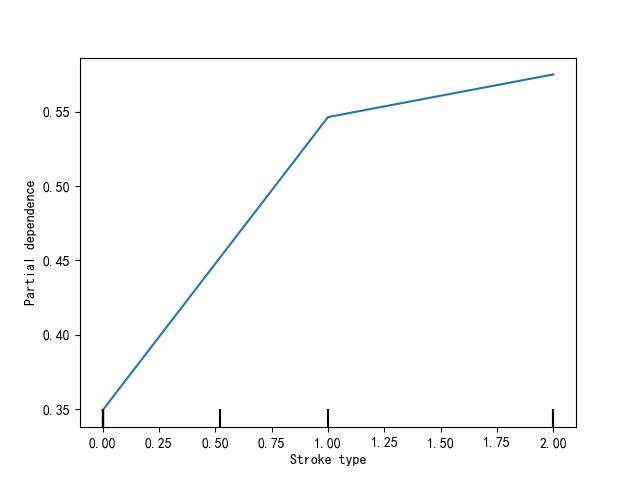

Supplement: Supplementary file 1 [file DataSheet1.zip › Supplementary Figure/Figure 5. Stroke type.png]
